# The episodic ataxia type 1 mutation I262T alters voltage-dependent gating and disrupts protein biosynthesis of human Kv1.1 potassium channels

**DOI:** 10.1038/srep19378

**Published:** 2016-01-18

**Authors:** Szu-Han Chen, Ssu-Ju Fu, Jing-Jia Huang, Chih-Yung Tang

**Affiliations:** 1 Department of Physiology, College of Medicine, National Taiwan University, Taipei, Taiwan; 2 Graduate Institute of Brain and Mind Sciences, College of Medicine, National Taiwan University, Taipei, Taiwan

## Abstract

Voltage-gated potassium (Kv) channels are essential for setting neuronal membrane excitability. Mutations in human Kv1.1 channels are linked to episodic ataxia type 1 (EA1). The EA1-associated mutation I262T was identified from a patient with atypical phenotypes. Although a previous report has characterized its suppression effect, several key questions regarding the impact of the I262T mutation on Kv1.1 as well as other members of the Kv1 subfamily remain unanswered. Herein we show that the dominant-negative effect of I262T on Kv1.1 current expression is not reversed by co-expression with Kvβ1.1 or Kvβ2 subunits. Biochemical examinations indicate that I262T displays enhanced protein degradation and impedes membrane trafficking of Kv1.1 wild-type subunits. I262T appears to be the first EA1 mutation directly associated with impaired protein stability. Further functional analyses demonstrate that I262T changes the voltage-dependent activation and Kvβ1.1-mediated inactivation, uncouples inactivation from activation gating, and decelerates the kinetics of cumulative inactivation of Kv1.1 channels. I262T also exerts similar dominant effects on the gating of Kv1.2 and Kv1.4 channels. Together our data suggest that I262T confers altered channel gating and reduced functional expression of Kv1 channels, which may account for some of the phenotypes of the EA1 patient.

A vast variety of different voltage-gated K^+^ (Kv) channels play critical roles in setting neuronal excitability, controlling neuronal firing frequencies, shaping action potential waveforms, and modulating neurotransmitter release^1^,^2^. Based on the amino acid sequences of pore-forming α subunits, Kv channels are grouped into 12 major subfamilies (Kv1-Kv12)^3^. A functional Kv channel is a homo- or hetero-tetramer comprising four α subunits^4^,^5^. Normally, only members of the same Kv channel subfamily may co-assemble to form functional heterotetramers^6^,^7^,^8^,^9^,^10^. In Kv1 channels, for example, this subfamily-specific tetramerization requires specific inter-subunit associations via recognition/stabilization sequences located in the cytoplasmic amino-terminal tetramerization domain^8^,^10^,^11^.

In neurons, α subunits of Kv1.1, a member of the Kv1 subfamily, co-assemble with auxiliary Kvβ subunits that confer fast inactivation gating and facilitate channel biosynthesis^12^,^13^. In addition, Kv1.1 α subunits may form heterotetramers with other isoforms of the Kv1 subfamily, including Kv1.2 and Kv1.4 subunits; this co-assembly of different Kv1 isoforms contributes to the functional diversity of Kv1 channels in various brain regions^13^. Since the subcellular localization of Kv1.1 α subunits mainly involves axon initial segments, juxtaparanodes at nodes of Ranvier, and presynaptic axon terminals^14^,^15^, the modulation of Kv1.1 biophysical properties by Kvβ, Kv1.2, or Kv1.4 subunits is essential for shaping key neuronal features such as dynamic neurotransmitter release patterns in response to different action potential firing frequencies^16^,^17^.

Episodic ataxia type 1 (EA1) is an autosomal dominant and sporadic neurological disorder characterized by frequent, short-lasting attacks of uncoordinated movements and involuntary, repetitive muscle contraction (myokymia); genetic analyses indicate that EA1 is associated with mutations in the *KCNA1* gene on chromosome 12p that encodes the human Kv1.1 α subunit^18^,^19^,^20^,^21^,^22^. To date, over 25 different EA1 mutations have been identified, with most being missense mutations^22^,^23^,^24^. Despite the presence of a significant disparity in functional phenotypes, the majority of EA1-related mutant Kv1.1 α subunits is associated with a loss of channel function or a change in the biophysical property, and the mutant subunits usually exert dominant-negative effects on their wild-type (WT) counterpart^19^,^25^,^26^,^27^,^28^. In general, however, no clear correlation can be established between the clinical phenotypes of EA1 patients and the locations/types of Kv1.1 mutations^20^,^22^,^28^,^29^,^30^.

I262T, an EA1-associated mutation of a highly conserved residue at the S3 transmembrane segment of the Kv1.1 α subunit, was originally identified from a 10-year-old girl with atypical phenotypes such as distal weakness, paresis of foot extensors, and prolonged limb stiffness (neuromyotonia) lasting up to 12 hours^31^. A previous biophysical characterization reveals that the I262T mutant displays decreased K^+^ currents, reduced surface protein level, and significant dominant-negative effect on the functional expression of Kv1.1 WT channels^32^. Nevertheless, several key questions regarding I262T remain unanswered. For example, it is unknown whether I262T affects voltage-dependent activation and inactivation of Kv1.1. Nor is it clear how the mutation disrupts protein biosynthesis to manifest reduced surface expression of Kv1.1 channels. Moreover, it remains to be determined regarding the effect of I262T on the gating of other isoforms of the Kv1 subfamily. To address these critical issues, herein we study the functional and biochemical properties of the I262T mutant in the absence or presence of different Kv1 α and Kvβ subunits. Our data suggest that the pathophysiological impact of the mutation entails both altered gating property and defective protein biosynthesis of Kv1.1 channels.

## Results

### I262T alters voltage-dependent activation of Kv1.1 channels

**Figure 1:**
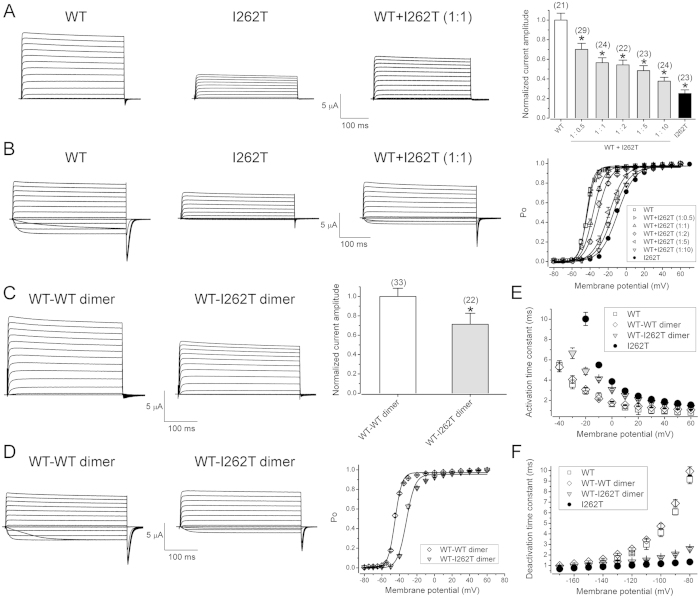
I262T exerts dominant effects on functional expression and voltage-dependent gating of Kv1.1 channels. Functional characterization of the I262T mutant in *Xenopus* oocytes. (**A**) Dominant-negative effects of I262T. (*Left*) Representative current traces of Kv1.1 WT, I262T, and equal-molar co-expression of WT and I262T [WT + I262T (1:1)]. In all oocyte injection conditions hereafter, the cRNA concentration is 0.5 μg/μl for each construct. The external bath solution contains 3 mM KCl. The holding potential is −90 mV. The voltage protocol comprises a 370-ms test potential (ranging from −80 mV to + 60 mV in + 10 mV steps) followed by −90-mV tail potential. (*Right*) Normalized peak current amplitudes at the + 60-mV test potential. In the co-expression group (WT + I262T), the molar ratio of the mutant was increased from 0.5 up to 10. Data were normalized with respect to the average values of Kv1.1 WT recorded from the same batch of oocytes on the same day. Asterisks denote a significant difference from the WT control (*, *t*-test: p < 0.05). (**B**) I262T shifts the voltage-dependence of Kv1.1. (*Left*) Representative current traces in the external solution containing 60 mM KCl. (*Right*) Steady-state activation (*P*_*o*_–V) curves derived from isochronal tail currents at −90 mV in response to various test pulse potentials. (**C**) Representative current traces in 3-mM KCl bath solution (*left*) and normalized peak current amplitudes (*right*) of Kv1.1 WT-WT dimer and WT-I262T dimer. Asterisks denote a significant difference from the WT-WT dimer control (*, *t*-test: p < 0.05). (**D**) Representative current traces in 60-mM KCl bath solution (*left*) and *P*_o_–V curves (*right*) of WT-WT and WT-I262T dimers. (**E,F**) I262T slows activation kinetics but speeds deactivation kinetics of Kv1.1 channels. Activation and deactivation time constants were derived from one-exponential fits of the rising phase of test currents (3 mM KCl) and tail currents (60 mM KCl), respectively. See Supplementary Table S1 for more details on *P*_o_–V parameters.

Consistent with the prior findings in Pro-5 cells by Zhu *et al*.^32^, we observed that, upon heterologous expression in *Xenopus* oocytes, the I262T mutant exhibits defective K^+^ current amplitude and exerts significant dominant-negative effect on the functional expression of Kv1.1 WT in a concentration-dependent manner (Fig. 1A). Therefore, the first question we asked was whether the EA1-associated mutation affects the voltage-dependent gating properties of Kv1.1 channels. By using 60 mM KCl in the external bath solution, we noticed that the I262T mutant displays negligible inward K^+^ currents during the depolarizing test pulses ranging from −60 to −20 mV (Fig. 1B), suggesting the presence of a sizable change in the voltage dependence of the mutant channel. We thus analyzed the steady-state voltage-dependence of Kv1.1 open probability (the *P*_o_–V curve), which is derived from fitting the tail current amplitudes at −90 mV with the Boltzmann equation. Figure 1B and Supplementary Table S1 demonstrate that the I262T mutation leads to a dramatic change of the half-maximal voltage (half-activation point) from about −43.2 to −9.6 mV, and a substantial increase of the slope factor from about 4.4 to 9.4. Moreover, upon co-expressing different ratios of WT and mutant subunits in oocytes, we found that I262T produces a dominant effect on the voltage activation of WT Kv1.1 in a concentration-dependent manner (Fig. 1B; Suppl. Table S1).

Next we constructed Kv1.1 tandem dimers consisting of two WT subunits (WT-WT dimer), or WT and I262T subunits (WT-I262T dimer). Figure 1C,D illustrate that, compared to the WT-WT dimer, the WT-I262T dimer displays reduced K^+^ current amplitude and right-shifted Po-V curve, similar to the effects of equal-molar co-expression of WT and I262T subunits. These results are consistent with the idea that the preceding dominant effects of I262T on steady-state current amplitude and voltage-dependence of Kv1.1 WT channels indeed reflect inter-subunit interactions within WT-I262T heterotetramers. In addition, both I262T homotetramers and WT-I262T heterotetramers show slower activation kinetics but faster deactivation kinetics (Fig. 1E,F), suggesting that the I262T mutation may also modify the time course of Kv1.1 channel gating.

### I262T shows enhanced protein degradation and disrupts membrane trafficking of Kv1.1 WT channels

**Figure 2:**
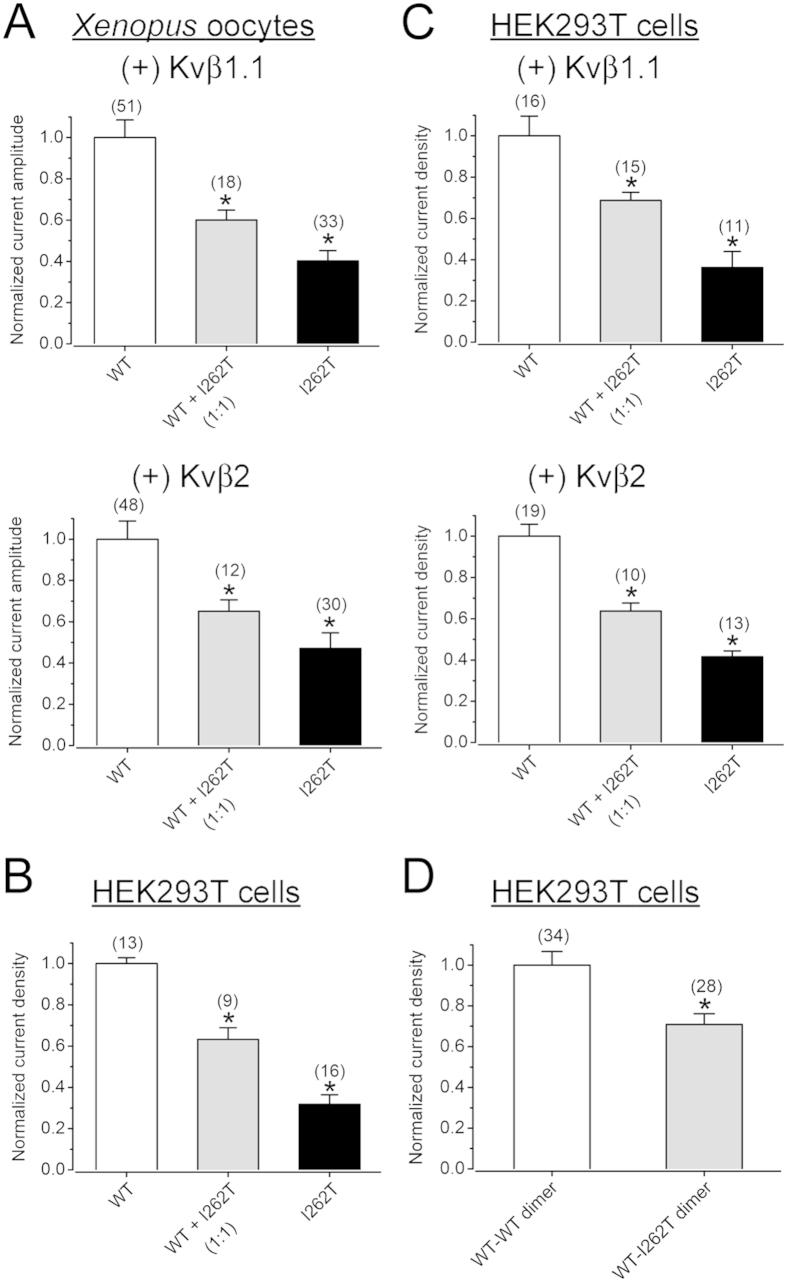
Co-expression with Kvβ subunits does not reverse the current suppression effect of I262T. (**A**) Normalized peak current amplitudes (at + 60 mV) in *Xenopus* oocytes. Despite the presence of Kvβ1.1 or Kvβ2 subunits, 262T displays significant dominant-negative effects. (**B,C**) Normalized peak current densities (at + 60 mV) in HEK293T cells. I262T shows comparable current suppression effects in the absence or presence of Kvβ subunits. (**D**) Normalized peak current densities (at + 60 mV) of Kv1.1 WT-WT dimer and WT-I262T dimer in HEK293T cells. The voltage protocol is the same as that in Fig. 1A. Asterisks denote a significant difference from the corresponding WT control (*, *t*-test: p < 0.05). Kv1.1α and Kvβ subunits were co-expressed in the molar ratio 1:5. See Supplementary Figs S1 and S2 for representative current traces.

Although the decreased functional expression of I262T was previously attributed to a reduced surface abundance of the K^+^ channel^32^, it remains unclear how the mutation disrupts protein biosynthesis of Kv1.1 channels. The biosynthesis of Kv1.1 α subunits is known to be facilitated by co-assembly with auxiliary Kvβ subunits^12^,^13^. We therefore asked whether the suppression effect of I262T on Kv1.1 current amplitude may be affected by the facilitating effect of Kvβ subunits. Figure 2A and Supplementary Fig. S1 depict that, in *Xenopus* oocytes, co-expressing with Kvβ1.1 or Kvβ2 subunits did not either rescue the mutant’s inherent deficiency or reverse its dominant-negative effects. This observation suggests that the facilitating effect of Kvβ subunits does not disproportionally enhance I262T current level and is therefore insufficient to counterbalance the biosynthetic disruption conferred by the mutation.

**Figure 3:**
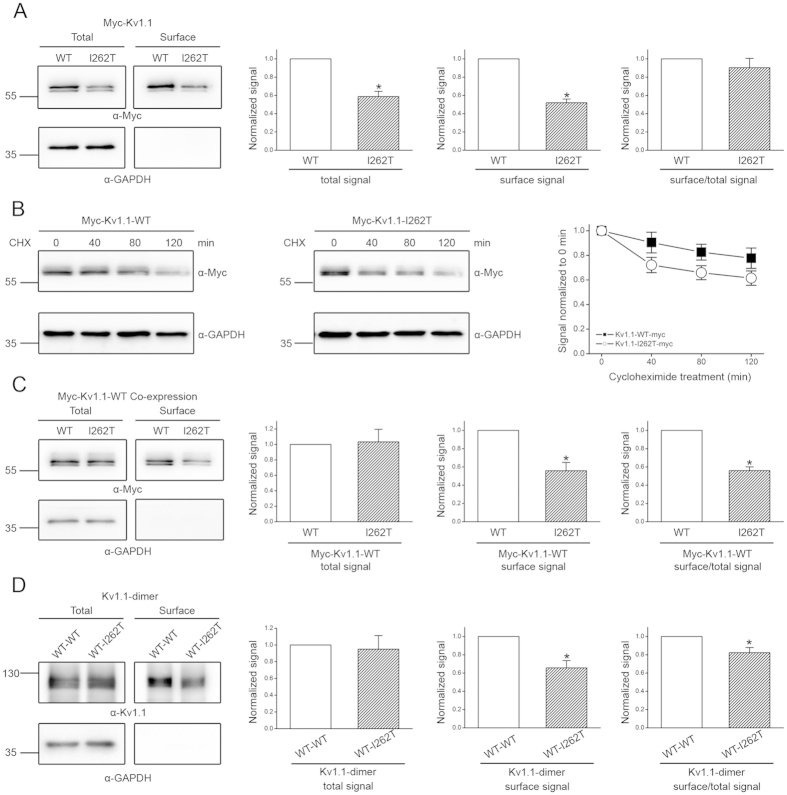
Biochemical analyses of the mechanism underlying the dominant-negative effect of I262T. (**A**) Surface biotinylation analyses of Myc-tagged Kv1.1 (Myc-Kv1.1) WT and I262T in HEK293T cells. (*Left*) Representative immunoblots. The molecular weight markers (in kilodaltons) are labeled to the left, and the immunoblotting antibodies (α-Myc and α-GAPDH) are specified below the immunoblots. Cell lysates from biotinylated intact cells were either directly employed for immunoblotting analyses (total) or subject to streptavidin pull-down before being used for immunoblotting analyses (surface). (*Right*) Quantification of total protein level (total signal), surface protein level (surface signal), and surface expression efficiency (surface/total signal). I262T shows reduced protein level. The total protein density was standardized as the ratio of total Myc signal to the signal of the loading control GAPDH. The surface protein density was standardized as the ratio of surface Myc signal to the cognate total GAPDH signal. The efficiency of surface presentation is expressed as surface protein density divided by the corresponding standardized total protein density. (**B**) The kinetics of Myc-Kv1.1-WT and Myc-Kv1.1-I262T protein degradation in the presence of 100 μg/ml cycloheximide (CHX) treatments of different durations. (*Left*) Representative immunoblots. (*Right*) Quantification of Kv1.1 protein degradation time course. Protein densities were standardized as the ratio of Kv1.1 signals to the cognate GAPDH signals, followed by normalization to those of the corresponding control at 0 hr. See Supplementary Fig. S3 for details on semi-logarithmic linear-regression analyses of the degradation time course. (**C**) Surface biotinylation analyses of Myc-Kv1.1-WT co-expressed with untagged WT or I262T (1:1 molar ratio). (**D**) Surface biotinylation analyses of Kv1.1 WT-WT dimer and WT-I262T dimer. Kv1.1 dimers were detected with the anti-Kv1.1 (αKv1.1) antibody. Asterisks denote significant difference from the WT control (*, *t*-test: p < 0.05; n = 3–6). The gels were run under the same experimental conditions. Uncropped images of immunoblots are shown in Supplementary Fig. S5.

To directly address the biosynthetic mechanism underlying the reduced Kv1.1 functional expression imposed by I262T, we decided to study the protein expression of Myc-tagged Kv1.1 channels in HEK293T cells, wherein the mutant also displays defective current level and notable dominant-negative effects (Fig. 2B–D; Suppl. Fig. S2). We began by performing surface biotinylation experiments. Figure 3A shows that, compared to Kv1.1 WT, I262T is indeed associated with decreased protein abundance in the membrane surface. Nevertheless, we also noticed a comparable reduction (~45%) in the total protein signal for I262T. Furthermore, no significant difference in the surface/total protein signal ratio was observed between Kv1.1 WT and the mutant (Fig. 3A), implying that I262T does not appear to be defective in its membrane trafficking efficiency, and that the observed reduced surface expression may instead result from enhanced protein degradation of the mutant channel. To directly test this hypothesis, we went on to determine the protein half-life of Kv1.1 channels by performing cycloheximide chase experiments. Figure 3B and Supplementary Fig. S3 illustrate that the protein half-time of the WT is about 4.1 hours, which is in agreement with the value (4.2 hours) previously derived from pulse-chase experiments^33^. By contrast, the protein half-time of I262T is only about 2.8 hrs, consistent with the idea that the EA1 mutant is associated with enhanced protein degradation, thereby manifesting reduced surface expression and smaller K^+^ current amplitude.

The next question we asked was whether a similar biosynthetic scenario may account for the dominant-negative effect of I262T on its WT counterpart. To address this issue, Myc-tagged Kv1.1 WT was co-expressed with untagged I262T in HEK293T cells, followed by immunoblotting with the anti-Myc antibody. Figure 3C demonstrates that the mutant fails to measurably change the total signal of the WT protein, implying that the dominant-negative effect cannot be attributed to enhanced protein degradation of Kv1.1 WT. Co-expression with I262T, however, leads to substantially reduced (~40%) surface expression of the WT channel. We also repeated the same experiments by comparing WT-WT dimers and WT-I262T dimers. Figure 3D shows that, despite the presence of similar total protein signal, the cell surface expression of WT-I262T dimers decreases by about 40%. Together these data suggest that, unlike I262T homotetramers, co-assembly of the mutant subunit with Kv1.1 WT results in impaired membrane trafficking of the heterotetramers.

### I262T changes voltage-dependent inactivation of Kv1.1 channels

**Figure 4:**
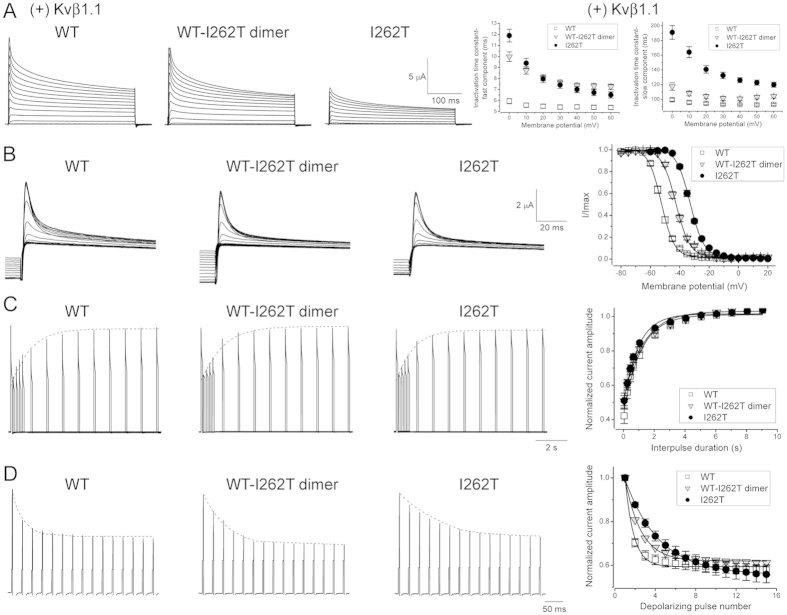
I262T alters Kvβ1.1-mediated voltage-dependent inactivation of Kv1.1 channels. (**A**) (*Left*) Representative Kv1.1 current traces of WT, WT-I262T dimer, or I262T co-expressed with Kvβ1.1 (molar ratio 1:5) in *Xenopus* oocytes. The voltage protocol is the same as that in Fig. 1A. The external solution contains 3 mM KCl. (*Right*) I262T decelerates the fast inactivation kinetics of Kv1.1 channels. Inactivation time constants were derived from two-exponential fits of the falling phase of test currents. (**B**) I262T shifts the voltage-dependent inactivation of Kv1.1. (*Left*) Representative current traces in response to the voltage protocol comprising a 350-ms depolarizing prepulse potential (ranging from −80 mV to + 20 mV in + 5 mV steps), followed by a test pulse (fixed at + 60 mV) for 100 ms. (*Right*) Steady-state inactivation curves derived from the normalization of peak current amplitudes (at the + 60-mV test pulse; I/I_max_) in response to different prepulse potentials. See Supplementary Table S1 for more details on the parameters of the inactivation curves. (**C**) Lack of effect of I262T on the inactivation recovery of Kv1.1. (*Left*) Representative current traces in response to a double-pulse voltage protocol comprising two consecutive 100-ms + 40-mV test pulses that were separated by −90- mV interpulses of increasing durations (from 0.05 to 9 sec). The dotted line denotes the recovery time course of the K^+^ current in response to the second test pulse. (*Right*) Normalized peak current amplitudes (at the second + 40-mV pulse) as a function of interpulse duration. (**D**) I262T changes the cumulative inactivation of Kv1.1. (*Left*) Representative current traces in response to a 40-Hz train of 3-ms + 40-mV test pulses with 25-ms interpulse intervals (holding at −90 mV). No –P/4 leak subtraction was performed. The dotted line denotes the time course of K^+^ current reduction in response to 15 consecutive test pulses. (*Right*) Normalized peak current amplitudes (at + 40 mV) as a function of test pulse number. See Supplementary Table S2 for more details on the kinetics of inactivation recovery and cumulative inactivation.

It remains unknown whether the I262T mutant may impair fast inactivation gating of Kv1.1 channels, which is conferred by co-assembly with the auxiliary subunit Kvβ1, but not Kvβ2 (Suppl. Figs S1 and S2)^12^. To address this important question, we co-expressed Kvβ1.1 with Kv1.1 in *Xenopus* oocytes. Figure 4A illustrates that, compared to Kv1.1 WT, both I262T homotetramers and WT-I262T heterotetramers display decelerated inactivation kinetics, indicating that the EA1 mutation may hinder the rate of entry into Kvβ1.1-mdediated Kv1.1 inactivation. Moreover, the voltage-dependence of fast inactivation (the steady-state inactivation curve) for I262T homotetramers and WT-I262T heterotetramers is right-shifted by about 20 and 11 mV, respectively (Fig. 4B; Suppl. Table S1), reminiscent of the effect of the mutation on the steady-state activation curve (Fig. 1B; Suppl. Table S1). By contrast, although the EA1 mutation effectively increases the slope factors of Kv1.1 activation curves, neither I262T homotetramers nor WT-I262T heterotetramers show notable change in the slope factor of their inactivation curves. This differential effect on the slope factor suggests that the mutation at the S3 transmembrane segment may instigate a partial uncoupling of voltage-dependent activation and inactivation in Kv1.1 channels.

We also examined the recovery from inactivation (refractory period) of these channels by employing a double-pulse protocol (Fig. 4C), wherein two consecutive 100-ms + 40-mV depolarizing test pulses are separated by interpulse intervals (holding at −90 mV) of increasing durations (from 0.05 to 9 sec). By plotting the relative amount of inactivation (observed at the second test pulse) as a function of the interpulse duration, we found that the I262T mutation does not apparently modify the time course of inactivation recovery for Kv1.1 channels (Fig. 4C; Suppl. Table S2).

Since enhanced action potential frequency may increase the extent of cumulative (use-dependent) inactivation in Kv1.1 channels, the recruitment of I262T homotetramers or WT-I262T heterotetramers during high-frequency neuronal firing may be affected by their slower onset of Kvβ1.1-mediated inactivation. To address this possibility, we mimicked a burst of action potentials by applying a 40-Hz train of 3-ms + 40-mV test pulses with 25-ms interpulse intervals (holding at −90 mV). Figure 4D and Supplementary Table S2 illustrate that both I262T homotetramers and WT-I262T heterotetramers exhibit slower cumulative inactivation kinetics, suggesting that I262T may interfere with the repriming of Kv1.1 channels during a train of action potentials.

### I262T modifies voltage-dependent activation and inactivation of Kv1.4 channels

**Figure 5:**
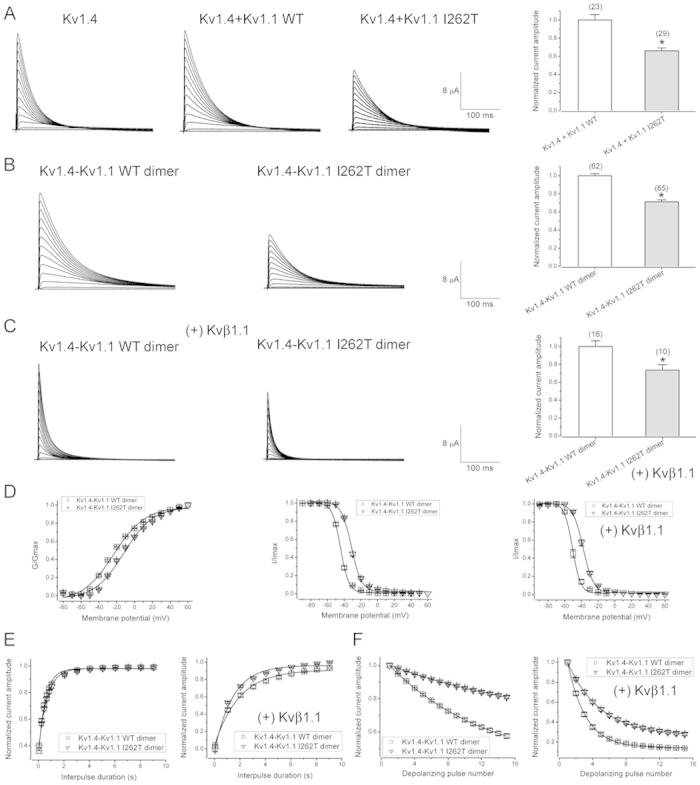
I262T exhibits dominant effects on voltage-dependent gating of Kv1.4 channels. Co-expression of Kv1.4 and Kv1.1 I262T in *Xenopus* oocytes. (**A**) Kv1.4 fails to affect the defective expression of I262T. (*Left*) Representative current traces of Kv1.4 WT alone (Kv1.4), co-expression of Kv1.4 WT and Kv1.1 WT (Kv1.4 + Kv1.1 WT), and co-expression of Kv1.4 WT and Kv1.1 I262T (Kv1.4 + Kv1.1 I262T). The inherent fast inactivation kinetics of Kv1.4 decelerates in the presence of Kv1.1. The voltage protocol is the same as that in Fig. 1A. The external solution contains 3 mM KCl. (*Right*) Normalized current amplitudes (at + 60 mV) for the two Kv1.4 + Kv1.1 co-expression conditions. (**B**) Representative current traces (*left*) and normalized current amplitudes (*right*) for Kv1.4-Kv1.1 WT dimer and Kv1.4-Kv1.1 I262T dimer. (**C**) Representative current traces (*left*) and normalized current amplitudes (*right*) for the two Kv1.4-Kv1.1 dimers in the presence of Kvβ1.1 (molar ratio 1:5). Asterisks denote a significant difference from the WT control (*, *t*-test: p < 0.05). (**D**) I262T shifts the voltage-dependent activation (*left*) and inactivation (*center, right*) of Kv1.4. Steady-state activation curves of the two Kv1.4-Kv1.1 dimers were expressed as normalized peak current amplitudes in response to different test pulse potentials. Steady-state inactivation curves in the absence or presence of Kvβ1.1 were obtained by employing the same voltage protocol as described in Fig. 4B. See Supplementary Table S3 for more details on the parameters of the activation and inactivation curves. (**E,F**) I262T does not prominently affect the inactivation recovery but significantly slowed the cumulative inactivation of Kv1.4. Normalized peak current amplitudes (with or without Kvβ1.1) of the two Kv1.4-Kv1.1 dimers were plotted against interpulse duration (**E**) and test pulse number (**F**) as described in Fig. 4C,D, respectively. See Supplementary Table S4 for more details on the kinetics of inactivation recovery and cumulative inactivation.

In neurons, the functional diversity of Kv1 channels is exemplified by the co-assembly of Kv1.1 with other isoforms of the Kv1 subfamily, including Kv1.2 and Kv1.4. It is still unclear whether the I262T mutant may affect the voltage-dependent gating of other Kv1 subfamily members. Figure 5A depicts that, in *Xenopus* oocytes, the reduced functional expression of Kv1.1 I262T is not reversed by co-expression with Kv1.4. A similar lack of effect was also observed when we co-expressed the mutant with Kv1.2 (Suppl. Fig. S4A). Moreover, by examining the functional expression of Kv1.4-Kv1.1 WT dimers and Kv1.4-Kv1.1 I262T dimers, we noticed that the current amplitude of the Kv1.4-Kv1.1 I262T heterotetramer is also significantly smaller than that of the Kv1.4-Kv1.1 WT heterotetramer (Fig. 5B), which is not visibly affected by co-expression with the auxiliary Kvβ1.1 subunit (Fig. 5C).

Figure 5D and Supplementary Table S3 show that, compared to its WT counterpart, the steady-state activation curve of the Kv1.4-Kv1.1 I262T heterotetramer shows a depolarizing shift of about 10 mV, indicating that the EA1 mutant is capable of modifying the voltage-dependent activation of Kv1.4. Likewise, we also observed a similar shift in the Po-V curve when we co-expressed the mutant with Kv1.2 (Suppl. Fig. S4B). Kv1.4 manifests inherent fast inactivation gating that is notably accelerated by co-expression with the Kvβ1.1 subunit (Fig. 5A–C)^34^. In the presence of I262T, the steady-state inactivation curves of both inherent and Kvβ1.1-mediated Kv1.4 inactivation are right-shifted by more than 10 mV (Fig. 5D; Suppl. Table S3). The slope factors for steady-state activation and inactivation curves of Kv1.4, however, are not measurably altered by the Kv1.1 mutant (Suppl. Table S3).

Regarding the kinetic properties of Kv1.4 inactivation, I262T does not appear to affect either the inherent or the Kv1.1β-mediated inactivation kinetics of Kv1.4 (Fig. 5B,C). In addition, the EA1 mutant does not seem to have a pronounced effect on the time course of recovery from inactivation for Kv1.4 (Fig. 5E; Suppl. Table S4). Nonetheless, the decay time constant of cumulative inactivation for Kv1.4 is significantly increased in the presence of the mutant (Fig. 5F; Suppl. Table S4), suggesting that I262T may hinder the use-dependent inactivation of Kv1.4 during high-frequency firing of action potentials.

## Discussion

**Figure 6:**
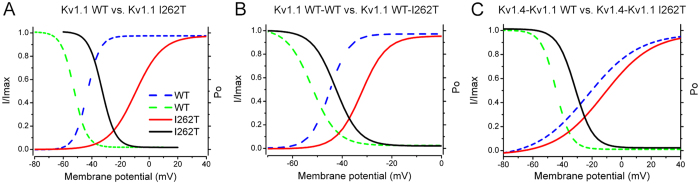
I262T modifies the window currents of Kv1.1 and Kv1.4 channels. Steady-state activation and inactivation curves of “Kv1.1 vs. I262T” (**A**), “Kv1.1 WT-WT dimer vs. WT-I262T dimer” (**B**), and “Kv1.4-Kv1.1 WT dimer vs. Kv1.4-Kv1.1 I262T dimer” (**C**) were assembled from those shown in Figs 1,4 and 5. Kv1.1 inactivation curves are based on Kvβ1.1-mediated fast inactivation, whereas Kv1.4-Kv1.1 inactivation curves concern the inherent fast inactivation of Kv1.4. Solid lines refer to constructs comprising the mutant, whereas dashed lines denote the corresponding WT control. The window current of each indicated construct is defined by the triangular area underneath the overlap of activation and inactivation curves. See Supplementary Table S5 for more details on the parameters of window currents.

Here, we provide a series of different evidence showing that the EA1-assoicated I262T mutation may prominently modify the voltage-dependent gating properties of Kv1 channels. The overlapping area of steady-state activation and inactivation curves represents the range of voltage in which a maximal number of inactivating ion channels remains tonically active, also known as window currents. In direct contrast with its dramatic enhancement of the slope factor of the steady-state activation curve, I262T does not visibly alter the slope factor of the Kvβ1-mediated inactivation curve; moreover, I262T exerts a significantly larger right-shift of the activation curve (Suppl. Table S1). Therefore, compared to their WT counterparts, I262T homotetramers exhibit a depolarizing shift of Kv1.1 window currents by more than 20 mV, as well as a reduction of the window current area by about 30% (Fig. 6A; Suppl. Table S5). Interestingly, these observations strongly argue that the I262T mutation at the S3 segment may instigate an uncoupling of voltage-dependent activation and inactivation in Kv1.1 channels. Two EA1-like mutations (one at the S2 and the other at the S6) have also been shown to uncouple inactivation gating from activation gating in Shaker K^+^ channels^35^. The mechanism underlying these uncoupling effects is currently unknown. By comparing WT-WT dimers and WT-I262T dimers, we also infer that the EA1 mutation may alter the voltage range and the area of Kv1.1 window currents by about 10 mV and 20%, respectively (Fig. 6B; Suppl. Table S5). Concerning Kv1.4-Kv.1.1 WT dimers and Kv1.4-Kv.1.1 I262T dimers, I262T is expected to shift Kv1.4-Kv1.1 widow currents by about 13 mV toward the depolarizing direction, as well as increasing the window current area by about 40% (Fig. 6C; Suppl. Table S5). As to be explained below, the aforementioned modifications of Kv1.1/Kv1.4 window currents may have a profound impact on the membrane excitability of neurons.

In this study, we present direct biochemical evidence showing that the I262T mutation induces significant disruption of the protein biosynthesis of Kv1.1 channels. I262T homotetramers display notably reduced protein level and remarkably shorter protein half-time, both of which are consistent with the idea that the EA1 mutant is associated with enhanced protein degradation. Our inference, however, is in direct contrast with the conclusion previously drawn by Zhu *et al*.^32^. Although both research groups employed surface biotinylation experiments to demonstrate reduced surface expression of I262T, Zhu *et al*. proposed that the Kv1.1 mutant is associated with defective membrane trafficking. They provided biochemical evidence suggesting that, compared to the WT, I262T is more abundant in the endoplasmic reticulum (ER). A closer look into the previous study reveals that Zhu *et al*. determined the amount of Kv1.1 protein in the ER by performing immunoprecipitation of an isolated ER membrane fraction with the anti-calnexin antibody. In other words, instead of directly comparing the relative abundance of the two Kv1.1 subunits in the ER, Zhu *et al*. were actually studying the channel proteins’ relative co-immunoprecipitation efficiency with calnexin. Calnexin is merely one of the numerous molecular chaperones responsible for protein folding in the ER. It is not clear to us whether their results really support the idea that I262T displays enhanced ER retention, or rather the data should be interpreted as an increased tendency of the mutant channel in forming protein complexes with calnexin. Therefore, the ratio of “biotinylated” to “calnexin-associated” fractions does not appear to be a fair parameter for evaluating the membrane trafficking property of Kv.1.1 channels. By contrast, herein we directly determined protein level from total cell lysates, and we employed the ratio of “biotinylated” to “total” fractions to quantify the membrane trafficking efficiency of Kv.1.1 channels. Our biochemical analyses suggest that Kv1.1 WT homotetramers and I262T homotetramers do not exhibit discernible difference in the membrane trafficking efficiency.

At least three EA1 mutations were previously associated with pronouncedly decreased protein level: the nonsense mutation R417X (at the carboxyl-terminus)^30^,^36^ and the missense mutations R239S and F249I (at the S2-S3 linker)^27^. Unlike the reduced but significant functional expression of I262T, the three EA1 mutants yield minimal or no detectable K^+^ currents^25^,^27^,^29^,^30^. Moreover, R239S was shown not to exert measurable dominant-negative effect on Kv1.1 WT^27^. To the best of our knowledge, I262T is the first EA1 mutant directly demonstrated to exhibit impaired protein stability. Based on *in silico* protein structure modeling, a replacement of the highly conserved I262 residue at the S3 transmembrane segment was predicted to induce subtle conformational change in the Kv1.1 α subunit, such as modifying the local conformation surrounding the voltage sensor region^32^. In addition to affecting voltage-dependent gating properties, we speculate that this subtle conformational change may also disrupt protein folding. We propose that this putative folding defect may render a sizable portion of the mutant channel unfavorable for the protein quality control system in the ER, thereby manifesting enhanced ER-associated protein degradation and reduced K^+^ current amplitude. On the other hand, our biochemical analyses suggest that, upon forming heterotetramers, I262T fails to affect the protein stability of its WT counterpart; instead the EA1 mutant impedes the membrane trafficking of WT/I262T heterotetramers. Consistent with previous findings for other EA1 mutants^30^,^34^,^36^,^37^,^38^, our data suggest that the defective biosynthesis of I262T cannot be rescued by co-expression with Kvβ1.1, Kvβ2, Kv1.2, or Kv1.4 subunits.

The I262T mutation was identified from a young patient showing up to 12 hours of neuromyotonia^31^, which reflects a prominent hyper-excitability of peripheral motor axons^39^,^40^,^41^. In the PNS, large myelinated axons, including motor axons, contain only Kv1.1, Kv1.2, and Kvβ2 subunits, but not Kv1.4 and Kvβ1; moreover, Kv1.1 and Kv1.2 channels are principally located at juxtaparanodes at nodes of Ranvier, but perhaps not at axon terminals, of peripheral myelinated fibers^12^,^13^,^14^,^15^,^42^. In other words, Kv1.1 and Kv1.2 channels do not display fast inactivation and are essential for the prevention of aberrant firing of action potentials in large myelinated nerves in the PNS^43^,^44^. In the present study, we demonstrate that the I262T mutation leads to a wide variety of different loss-of-function consequences for Kv1 channels, including 1) enhanced I262T protein degradation, 2) defective membrane trafficking of Kv1.1 WT, 3) reduced Kv1.1 current amplitudes, 4) altered steady-state voltage-dependence (right-shifted half-activation point and larger slope factor) of Kv1.1/1.2, and 5) slower activation kinetics but faster deactivation kinetics of Kv1.1 channels. The presence of the I262T mutant may therefore confer significantly enhanced membrane excitability and excessive action potential firing of motor axons, a pathophysiological scenario that can in part explain the prolonged neuromyotonia phenotype of the EA1 patient. Consistent with this idea, rodents with loss-of-function Kv1.1 missense mutations indeed display spontaneous motor axon firing and the myokymia/neuromyotonia phenotype^45^,^46^.

In a subset of CNS neurons, Kv1.1, Kv1.4, and Kvβ1 subunits co-localize in the initial axonal segment, juxtaparanodal region at nodes of Ranvier, and axon terminals^12^,^13^,^14^,^15^. The I262T mutation prominently changes the gating properties of Kv1.1 and Kv1.4 channels, including 1) depolarizing shift of activation curves, inactivation curves, and window currents, 2) slower fast inactivation (for Kv1.1) and cumulative inactivation kinetics, and 3) altered window current area (smaller and lager for Kv1.1 and Kv1.4, respectively). Depolarizing shift and smaller area of Kv1.1 window currents, as well as reduced Kv1.1 current amplitude, will likely imply a higher probability for neurons to fire action potentials. By contrast, slower cumulative Kv1.1/1.4 inactivation, as well as larger Kv1.4 window current area, will reduce the broadening of action potentials at axon terminals during repetitive neuronal firing^16^,^47^, thereby suppressing excessive neurotransmitter release. Hence, as pointed out previously^34^, it is rather difficult to determine the relative contribution of these opposing EA1 mutation effects on the overall membrane excitability of different CNS neurons.

Another issue further complicating the significance of I262T-mediated change in Kv1.1/1.4 inactivation concerns the differential localization of Kv1 channels in the CNS. In the mammalian brain, Kv1.1 seems to be separated into two main subpopulations: one associated with Kv1.2 and the other with Kv1.4; moreover, Kvβ2, but not Kvβ1, appears to be preferentially present in many neurons that co-express Kv1.1 and Kv1.2^12^,^13^. In the cerebellum, for example, Kv1.1, Kv1.2, and Kvβ2, in the absence of Kv1.4 and Kvβ1, co-localize in the axon terminals of basket cells, as well as some interneurons in the granule cell layer, which may reasonably account for the ataxia phenotype observed in EA1 patients^48^. On the other hand, in the hippocampus, Kv1.1, Kv1.4, and Kvβ1 co-assemble into heteromultimers in the axons within the excitatory circuitry, whereas Kv1.1, Kv1.2, and Kvβ2 form heteromeric channels in a different subset of neurons. Nevertheless, it remains controversial whether EA1 patients exhibit cognitive dysfunctions^20^,^49^,^50^. In spite of these uncertainties, emerging evidence in primates suggests the presence of reciprocal connections between cerebellum and basal ganglia (via thalamus and pons, respectively) that may explain Parkinson’s disease-related pathological changes in the cerebellum^51^,^52^. Furthermore, heteromultimeric channels comprising Kv1.1, Kv1.2, and Kvβ2 play a critical role in regulating the output of deep cerebellar nuclear neurons to thalamic targets^53^, and Kv1.1, Kv1.4, and Kvβ1 are robustly co-expressed in the efferent neurons (globus pallidus and substantia nigra) of basal ganglia^54^. Although it is still an open question regarding the role of the reciprocal neural network in cerebellum function, it would be worthwhile in the future to test the intriguing hypothesis that the effect of the I262T mutation on voltage-dependent Kv1.1/1.4 activation and inactivation may destabilize intrinsic neuronal excitability and induce network variability in this two-way communication between the two subcortical structures.

In conclusion, we demonstrate that the pathophysiological impact of the I262T mutation entails altered channel gating and defective protein biosynthesis, both of which raise imperative questions that call for further elucidation of the structural and functional roles of the S3 transmembrane segment in Kv1.1 channels. Moreover, the ER molecular network dictating Kv1.1 protein homeostasis remains obscure. Our results here highlight the clinical significance of identifying the protein machinery (*e.g.*, E3 ubiquitin ligases and molecular chaperones) mediating the ER quality control system of Kv1.1, as the new information may offer novel therapeutic directions for EA1 mutations associated with decreased Kv1.1 protein level.

## Methods

### cDNA constructs

WT and mutant human Kv1.1 cDNAs (kindly provided by Dr. Joanna Jen, University of California, Los Angeles, USA), as well as cDNA for human Kv1.1 WT-WT dimer (in the pGEMA vector; kindly provided by Dr. Mauro Pessia, University of Perugia, Italy), were subcloned into the pcDNA3.1-V5-His-TOPO or the pcDNA3.1-Myc vector (Invitrogen). To create the Kv1.1 WT-I262T heterodimer, the mutant sequence was introduced into the WT-WT dimer by using the *Sph*I-*Sph*I restriction sites. Monomeric human Kv1.4 and dimeric human Kv1.4-Kv1.1 WT cDNAs subcloned in the pBF vector were also kindly provided by Dr. Mauro Pessia. To create the Kv1.4-Kv1.1 I262T dimer, the mutant sequence was transferred to the Kv1.4-Kv1.1 WT dimer by using the *Not*I-*PflM*I restriction sites. All constructs were verified by DNA sequencing. Other cDNA constructs employed in this study include pGEMA-human Kv1.2 (kindly provided by Dr. Mauro Pessia), pcDNA3-human Kvβ1.1 (kindly provided by Dr. Olaf Pongs, Saarland University, Germany), and pcDNA1.1-rat Kvβ2 (kindly provided by Dr. James Trimmer, University of California, Davis, USA).

### Xenopus oocyte preparation and cRNA injection

Adult female *Xenopus laevis* (African Xenopus Facility, Knysna, South Africa) were anesthetized by immersion in Tricaine (1.5 g/l). Animal handling protocols are in accordance with the Guidelines for the Care and Use of Mammals in Neuroscience and Behavioral Research (National Research Council 2003) and were approved by the Institutional Animal Care and Use Committee (IACUC) of College of Medicine, National Taiwan University. To isolate *Xenopus* oocytes, ovarian follicles were removed from the frogs, cut into small pieces, washed in ND96 solution [(in mM) 96 NaCl, 2 KCl, 1.8 MgCl_2_, 1.8 CaCl_2_, and 5 HEPES, pH 7.5], and then incubated at room temperature with Ca^2+^ -free ND96 containing collagenase (2 mg/ml) on an orbital shaker (~200 rpm) for about 60–90 min to remove follicular membrane. After several washes with collagenase-free, Ca^2+^ -free ND96, oocytes were transferred to ND96. Stage V-VI oocytes were selected for cRNA injection.

Capped cRNAs were transcribed *in vitro* from linearized cDNAs with the mMessage mMachine T7 kit (Ambion). cRNA concentration was determined by spectrophotometry (NanoDrop ND-1000, Thermo). Kv1.1 cRNAs were diluted to 0.5 μg/μl for oocyte injection. The total volume of cRNA injection was 41.4 nl per oocyte. Injected oocytes were stored at 16 °C in ND96 solution supplemented with 50 mg/L gentamycin.

### Two-electrode voltage clamp in Xenopus oocytes

48–72 hrs after cRNA injection, oocytes were functionally assayed in a recording bath containing Ringer solution [(in mM): 115 NaCl, 3 KCl, 1.8 CaCl_2_, 10 HEPES, pH 7.2]. Where indicated, 60 mM KCl was used (by replacing NaCl) to record tail currents. Niflumic acid (0.5 mM) was added to the bath solution to minimize the contribution of endogenous Ca^2+^ -activated Cl^-^ currents. The volume of external bath solution was about 200 μl. An agarose bridge was used to connect the bath solution with a ground chamber (containing 3 M KCl) into which two ground electrodes were inserted. Borosilicate electrodes (0.1–1 MΩ) were filled with 3 M KCl. K^+^ currents through Kv1 channels were acquired with the conventional two-electrode voltage-clamp technique with an OC-725C oocyte clamp (Warner). Data were filtered at 1 kHz and digitized at 100 μs per point (10 kHz) using the Digidata 1332A/pCLAMP 8.2 data acquisition system (Molecular Devices). All recordings were performed at room temperature (20–22 °C). Leak currents arising from passive membrane properties were subtracted by using the –P/4 method provided in the pCLAMP system. To prevent voltage clamp errors due to excessive current amplitudes, only data from K^+^ channels with current amplitudes (at + 60 mV) no larger than 20 μA were selected for further analyses.

### Cell culture and DNA transfection

Human embryonic kidney (HEK) 293T cells were grown in Dulbecco’s modified Eagle’s medium (DMEM) [supplemented with 2 mM glutamine, 10% heat-inactivated fetal bovine serum (Hyclone), 100 units/ml penicillin, and 50 μg/ml streptomycin] and were maintained at 37 °C in a humidified incubator with 95% air and 5% CO_2_. Transient transfection was performed by using the Lipofectamine 2000 (LF2000) reagent (Invitrogen). Cells were plated onto 12-well plates (for biochemical experiments) or poly-D-lysine-coated coverslips in 24-well plates (for electrophysiological recordings) 24 hrs before transfection. cDNA constructs were incubated with LF2000 reagent for 20 min at room temperature, and DNA-lipofectamine diluted in Opti-MEM (Invitrogen) was added to culture wells. After 6-hr incubation at 37 °C, the medium was changed and the culture cells were maintained in the 37 °C incubator for 24–48 hrs before being used for biochemical or electrophysiological experiments. Where indicated, cycloheximide (Sigma) was applied to the culture medium 24 hrs after transfection.

### Whole-cell patch clamp in HEK293T cells

Cells co-transfected with the cDNA for pEGFP and Kv1.1 (molar ratio 1:10) were identified with an inverted fluorescence microscope (Leica-DM IRB). Recording electrodes were pulled by a PP-830 puller (Narashige), and display a resistance of 2–3 MΩ when filled with the pipette solution. The pipette solution contains (in mM): 140 KCl, 1 MgCl_2_, 10 EGTA, 10 HEPES, pH 7.2; while the bath solution comprises (in mM): 140 NaCl, 5 KCl, 1 CaCl_2_, 10 HEPES, pH 7.2. Data were acquired and digitized with Axopatch 200B and Digidata 1440A, respectively, via pCLAMP 10.2 (Molecular Devices). Cell capacitances were measured using the built-in functions of pCLAMP 10.2 and were compensated electronically with Axopatch 200B. Data were sampled at 10 kHz and filtered at 1 kHz. Leak currents were subtracted by using the –P/4 method. All recordings were performed at room temperature (20–22 °C).

### Immunoblotting

Cells were washed twice with ice-cold PBS [(in mM) 137 NaCl, 2.7 KCl, 4.3 Na_2_HPO_4_. 2H_2_O, 1.4 KH_2_PO_4_, pH 7.3] supplemented with 2 mM EDTA, and resuspended in a lysis buffer [(in mM) 150 NaCl, 5 EDTA, 50 Tris-HCl pH7.6, 1% Triton X-100) containing a protease inhibitor cocktail (Roche Applied Science). After adding the Laemmli sample buffer to the lysates, samples were sonicated on ice and heated at 70 °C for 5 min. Samples were then separated by 15% SDS-PAGE, electrophoretically transferred to nitrocellulose membranes, and detected using rabbit anti-GAPDH (1:5000; GeneTex), rabbit anti-Kv1.1 (1:2500; Upstate), or mouse anti-Myc (clone 9E10) antibodies. Blots were then exposed to horseradish peroxidase-conjugated anti-mouse/rabbit IgG (1:5000; Jackson ImmunoResearch), and revealed by an enhanced chemiluminescence detection system (Thermo Scientific). Results shown are representative of at least three independent experiments. Densitometric scans of immunoblots were quantified by using ImageJ (National Institute of Health).

### Biotinylation of cell surface proteins

Cells were washed extensively with D-PBS (Sigma) supplemented with 0.5 mM CaCl_2_, 2 mM MgCl_2_, followed by incubation in 1 mg/ml sulfo-NHS-LC-biotin (Thermo Scientific) in D-PBS at 4 °C for 1 hr with gentle rocking. Biotinylation was terminated by removing the biotin reagents and rinsing three times with 100 mM glycine in PBS, followed by once in TBS buffer [(in mM) 20 Tris-HCl, 150 NaCl, pH 7.4]. Solubilization was performed using a lysis buffer [(in mM) 150 NaCl, 50 Tris-HCl, 1% Triton X-100, 5 EDTA, 1 phenylmethylsulfonyl fluoride, pH 7.6] supplemented with the protease inhibitor cocktail. Insolubilized materials were removed by centrifugation. Solubilized cell lysates were incubated overnight at 4 °C with streptavidin-agarose beads (Thermo Scientific). Beads were washed once in the lysis buffer, followed by twice in a high-salt buffer [(in mM) 500 NaCl, 5 EDTA, 50 Tris-HCl, pH7.6, 0.1% Triton X-100] and once in a low-salt buffer [(in mM) 2 EDTA, 10 Tris-HCl, pH7.6, 0.1% Triton X-100]. The biotin-streptavidin complexes were eluted from the beads by heating at 70 °C for 5 min in the Laemmli sample buffer.

### Statistical analyses

All values are presented as mean ± SEM. Student’s *t*-test was employed to examine the significance of the difference between two means by using Origin 7.0 (Microcal Software).

## Additional Information

**How to cite this article**: Chen, S.-H. *et al*. The episodic ataxia type 1 mutation I262T alters voltage-dependent gating and disrupts protein biosynthesis of human Kv1.1 potassium channels. *Sci. Rep.* **6**, 19378; doi: 10.1038/srep19378 (2016).

## Supplementary Material

Supplementary Information

## References

[b1] Hille B. Ion channels of excitable membranes , (Sinauer, Sunderland, MA, 2001).

[b2] Johnston J., Forsythe I. D. & Kopp-Scheinpflug C. Going native: voltage-gated potassium channels controlling neuronal excitability. J Physiol 588, 3187–200 (2010). 20519310 10.1113/jphysiol.2010.191973 PMC2976014

[b3] Gutman G. A. . International Union of Pharmacology. LIII. Nomenclature and molecular relationships of voltage-gated potassium channels. Pharmacol Rev 57, 473–508 (2005). 16382104 10.1124/pr.57.4.10

[b4] MacKinnon R. Determination of the subunit stoichiometry of a voltage-activated potassium channel. Nature 350, 232–5 (1991). 1706481 10.1038/350232a0

[b5] Liman E. R., Tytgat J. & Hess P. Subunit stoichiometry of a mammalian K + channel determined by construction of multimeric cDNAs. Neuron 9, 861–71 (1992). 1419000 10.1016/0896-6273(92)90239-a

[b6] Covarrubias M., Wei A. A. & Salkoff L. Shaker, Shal, Shab, and Shaw express independent K + current systems. Neuron 7, 763–73 (1991). 1742024 10.1016/0896-6273(91)90279-9

[b7] Isacoff E. Y., Jan Y. N. & Jan L. Y. Evidence for the formation of heteromultimeric potassium channels in Xenopus oocytes. Nature 345, 530–4 (1990). 2112229 10.1038/345530a0

[b8] Li M., Jan Y. N. & Jan L. Y. Specification of subunit assembly by the hydrophilic amino-terminal domain of the Shaker potassium channel. Science 257, 1225–30 (1992). 1519059 10.1126/science.1519059

[b9] Deal K. K., Lovinger D. M. & Tamkun M. M. The brain Kv1.1 potassium channel: *in vitro* and *in vivo* studies on subunit assembly and posttranslational processing. J Neurosci 14, 1666–76 (1994). 8126562 10.1523/JNEUROSCI.14-03-01666.1994 PMC6577575

[b10] Shen N. V. & Pfaffinger P. J. Molecular recognition and assembly sequences involved in the subfamily-specific assembly of voltage-gated K + channel subunit proteins. Neuron 14, 625–33 (1995). 7695909 10.1016/0896-6273(95)90319-4

[b11] Shen N. V., Chen X., Boyer M. M. & Pfaffinger P. J. Deletion analysis of K + channel assembly. Neuron 11, 67–76 (1993). 8338669 10.1016/0896-6273(93)90271-r

[b12] Pongs O. & Schwarz J. R. Ancillary subunits associated with voltage-dependent K + channels. Physiol Rev 90, 755–96 (2010). 20393197 10.1152/physrev.00020.2009

[b13] Vacher H., Mohapatra D. P. & Trimmer J. S. Localization and targeting of voltage-dependent ion channels in mammalian central neurons. Physiol Rev 88, 1407–47 (2008). 18923186 10.1152/physrev.00002.2008 PMC2587220

[b14] Dodson P. D. & Forsythe I. D. Presynaptic K + channels: electrifying regulators of synaptic terminal excitability. Trends Neurosci 27, 210–7 (2004). 15046880 10.1016/j.tins.2004.02.012

[b15] Lai H. C. & Jan L. Y. The distribution and targeting of neuronal voltage-gated ion channels. Nat Rev Neurosci 7, 548–62 (2006). 16791144 10.1038/nrn1938

[b16] Geiger J. R. & Jonas P. Dynamic control of presynaptic Ca(2+) inflow by fast-inactivating K(+) channels in hippocampal mossy fiber boutons. Neuron 28, 927–39 (2000). 11163277 10.1016/s0896-6273(00)00164-1

[b17] Kole M. H., Letzkus J. J. & Stuart G. J. Axon initial segment Kv1 channels control axonal action potential waveform and synaptic efficacy. Neuron 55, 633–47 (2007). 17698015 10.1016/j.neuron.2007.07.031

[b18] Browne D. L. . Episodic ataxia/myokymia syndrome is associated with point mutations in the human potassium channel gene, KCNA1. Nat Genet 8, 136–40 (1994). 7842011 10.1038/ng1094-136

[b19] Rajakulendran S., Schorge S., Kullmann D. M. & Hanna M. G. Episodic ataxia type 1: a neuronal potassium channelopathy. Neurotherapeutics 4, 258–66 (2007). 17395136 10.1016/j.nurt.2007.01.010

[b20] Graves T. D. . Episodic ataxia type 1: clinical characterization, quality of life and genotype-phenotype correlation. Brain 137, 1009–18 (2014). 24578548 10.1093/brain/awu012 PMC3959554

[b21] Litt M. . A gene for episodic ataxia/myokymia maps to chromosome 12p13. Am J Hum Genet 55, 702–9 (1994). 7942848 PMC1918305

[b22] Baloh R. W. Episodic ataxias 1 and 2. Handb Clin Neurol 103, 595–602 (2012). 21827920 10.1016/B978-0-444-51892-7.00042-5

[b23] Tomlinson S. E. . Clinical, genetic, neurophysiological and functional study of new mutations in episodic ataxia type 1. J Neurol Neurosurg Psychiatry 84, 1107–12 (2013). 23349320 10.1136/jnnp-2012-304131 PMC4332158

[b24] D’Adamo M. C. . Novel phenotype associated with a mutation in the KCNA1(Kv1.1) gene. Front Physiol 5, 525 (2015). 25642194 10.3389/fphys.2014.00525 PMC4295438

[b25] Adelman J. P., Bond C. T., Pessia M. & Maylie J. Episodic ataxia results from voltage-dependent potassium channels with altered functions. Neuron 15, 1449–54 (1995). 8845167 10.1016/0896-6273(95)90022-5

[b26] D’Adamo M. C., Liu Z., Adelman J. P., Maylie J. & Pessia M. Episodic ataxia type-1 mutations in the hKv1.1 cytoplasmic pore region alter the gating properties of the channel. Embo J 17, 1200–7 (1998). 9482717 10.1093/emboj/17.5.1200 PMC1170468

[b27] Zerr P., Adelman J. P. & Maylie J. Episodic ataxia mutations in Kv1.1 alter potassium channel function by dominant negative effects or haploinsufficiency. J Neurosci 18, 2842–8 (1998). 9526001 10.1523/JNEUROSCI.18-08-02842.1998 PMC6792579

[b28] Tomlinson S. E., Hanna M. G., Kullmann D. M., Tan S. V. & Burke D. Clinical neurophysiology of the episodic ataxias: insights into ion channel dysfunction *in vivo*. Clin Neurophysiol 120, 1768–76 (2009). 19734086 10.1016/j.clinph.2009.07.003

[b29] Eunson L. H. . Clinical, genetic, and expression studies of mutations in the potassium channel gene KCNA1 reveal new phenotypic variability. Ann Neurol 48, 647–56 (2000). 11026449

[b30] Rea R., Spauschus A., Eunson L. H., Hanna M. G. & Kullmann D. M. Variable K(+) channel subunit dysfunction in inherited mutations of KCNA1. J Physiol 538, 5–23 (2002). 11773313 10.1113/jphysiol.2001.013242 PMC2290030

[b31] Klein A., Boltshauser E., Jen J. & Baloh R. W. Episodic ataxia type 1 with distal weakness: a novel manifestation of a potassium channelopathy. Neuropediatrics 35, 147–9 (2004). 15127317 10.1055/s-2004-817921

[b32] Zhu J., Alsaber R., Zhao J., Ribeiro-Hurley E. & Thornhill W. B. Characterization of the Kv1.1 I262T and S342I mutations associated with episodic ataxia 1 with distinct phenotypes. Arch Biochem Biophys 524, 99–105 (2012). 22609616 10.1016/j.abb.2012.05.006

[b33] Watanabe I., Zhu J., Recio-Pinto E. & Thornhill W. B. Glycosylation affects the protein stability and cell surface expression of Kv1.4 but Not Kv1.1 potassium channels. A pore region determinant dictates the effect of glycosylation on trafficking. J Biol Chem 279, 8879–85 (2004). 14688283 10.1074/jbc.M309802200

[b34] Imbrici P., D’Adamo M. C., Kullmann D. M. & Pessia M. Episodic ataxia type 1 mutations in the KCNA1 gene impair the fast inactivation properties of the human potassium channels Kv1.4-1.1/Kvbeta1.1 and Kv1.4-1.1/Kvbeta1.2. Eur J Neurosci 24, 3073–83 (2006). 17156368 10.1111/j.1460-9568.2006.05186.x

[b35] Boland L. M., Price D. L. & Jackson K. A. Episodic ataxia/myokymia mutations functionally expressed in the Shaker potassium channel. Neuroscience 91, 1557–64 (1999). 10391459 10.1016/s0306-4522(98)00718-0

[b36] Manganas L. N. . Episodic ataxia type-1 mutations in the Kv1.1 potassium channel display distinct folding and intracellular trafficking properties. J Biol Chem 276, 49427–34 (2001). 11679591 10.1074/jbc.M109325200

[b37] Imbrici P., D’Adamo M. C., Grottesi A., Biscarini A. & Pessia M. Episodic ataxia type 1 mutations affect fast inactivation of K + channels by a reduction in either subunit surface expression or affinity for inactivation domain. Am J Physiol Cell Physiol 300, C1314–22 (2011). 21307345 10.1152/ajpcell.00456.2010

[b38] Imbrici P. . A novel KCNA1 mutation identified in an Italian family affected by episodic ataxia type 1. Neuroscience 157, 577–87 (2008). 18926884 10.1016/j.neuroscience.2008.09.022

[b39] Gutmann L. & Libell D. When is myokymia neuromyotonia? Muscle Nerve 24, 151–3 (2001). 11180199 10.1002/1097-4598(200102)24:2<151::aid-mus10>3.0.co;2-7

[b40] Vincent A. Understanding neuromyotonia. Muscle Nerve 23, 655–7 (2000). 10797387 10.1002/(sici)1097-4598(200005)23:5<655::aid-mus1>3.0.co;2-e

[b41] Gutmann L. Myokymia and neuromyotonia 2004. J Neurol 251, 138–42 (2004). 14991346 10.1007/s00415-004-0331-5

[b42] Rasband M. N. . Distinct potassium channels on pain-sensing neurons. Proc Natl Acad Sci USA 98, 13373–8 (2001). 11698689 10.1073/pnas.231376298 PMC60878

[b43] Zhou L., Messing A. & Chiu S. Y. Determinants of excitability at transition zones in Kv1.1-deficient myelinated nerves. J Neurosci 19, 5768–81 (1999). 10407018 10.1523/JNEUROSCI.19-14-05768.1999 PMC6783064

[b44] Vabnick I. . Dynamic potassium channel distributions during axonal development prevent aberrant firing patterns. J Neurosci 19, 747–58 (1999). 9880595 10.1523/JNEUROSCI.19-02-00747.1999 PMC6782197

[b45] Brunetti O. . Kv1.1 knock-in ataxic mice exhibit spontaneous myokymic activity exacerbated by fatigue, ischemia and low temperature. Neurobiol Dis 47, 310–21 (2012). 22609489 10.1016/j.nbd.2012.05.002 PMC3402927

[b46] Ishida S. . Kcna1-mutant rats dominantly display myokymia, neuromyotonia and spontaneous epileptic seizures. Brain Res 1435, 154–66 (2012). 22206926 10.1016/j.brainres.2011.11.023

[b47] Giese K. P. . Reduced K + channel inactivation, spike broadening, and after-hyperpolarization in Kvbeta1.1-deficient mice with impaired learning. Learn Mem 5, 257–73 (1998). 10454353 PMC311244

[b48] Herson P. S. . A mouse model of episodic ataxia type-1. Nat Neurosci 6, 378–83 (2003). 12612586 10.1038/nn1025

[b49] Demos M. K. . A novel KCNA1 mutation associated with global delay and persistent cerebellar dysfunction. Mov Disord 24, 778–82 (2009). 19205071 10.1002/mds.22467

[b50] Gancher S. T. & Nutt J. G. Autosomal dominant episodic ataxia: a heterogeneous syndrome. Mov Disord 1, 239–53 (1986). 3504247 10.1002/mds.870010404

[b51] Hoshi E., Tremblay L., Feger J., Carras P. L. & Strick P. L. The cerebellum communicates with the basal ganglia. Nat Neurosci 8, 1491–3 (2005). 16205719 10.1038/nn1544

[b52] Bostan A. C., Dum R. P. & Strick P. L. The basal ganglia communicate with the cerebellum. Proc Natl Acad Sci USA 107, 8452–6 (2010). 20404184 10.1073/pnas.1000496107 PMC2889518

[b53] Ovsepian S. V. . A defined heteromeric KV1 channel stabilizes the intrinsic pacemaking and regulates the output of deep cerebellar nuclear neurons to thalamic targets. J Physiol 591, 1771–91 (2013). 23318870 10.1113/jphysiol.2012.249706 PMC3624850

[b54] Rhodes K. J. . Association and colocalization of the Kvbeta1 and Kvbeta2 beta-subunits with Kv1 alpha-subunits in mammalian brain K + channel complexes. J Neurosci 17, 8246–58 (1997). 9334400 10.1523/JNEUROSCI.17-21-08246.1997 PMC6573739

