# Supplementary material for: The episodic ataxia type 1 mutation I262T alters voltage-dependent gating and disrupts protein biosynthesis of human Kv1.1 potassium channels
Source: Sci Rep. 2016 Jan 18;6:19378. doi: 10.1038/srep19378 (PMC4726062; doi:10.1038/srep19378)

**Supplementary Information**

**The episodic ataxia type 1 mutation I262T alters voltage-dependent gating and disrupts protein biosynthesis of human Kv1.1 potassium channels**

Szu-Han Chen, Ssu-Ju Fu, Jing-Jia Huang, and Chih-Yung Tang

***Supplementary Table S1. Voltage-dependent activation and inactivation properties of human Kv1.1 WT and I262T channels.*** (related to Figures 1 and 4)

To generate steady-state activation (*P_o_*–V) curves, isochronal tail currents at -90 mV in response to various test pulse potentials were fit with a Boltzmann function: Po(V) = 1/{1+exp[(V_0.5a_-V)/*k_a_*]}, where V_0.5a_ is the half-maximal voltage for activation, and *k_a_* the slope factor. For steady-state Kvβ1.1-mediated inactivation curves, normalized peak current amplitudes (at +60-mV) in response to different prepulse potentials were fit with the Boltzmann function: Po(V) = 1/{1+exp[(V_0.5i_-V)/*k_i_*]}, where V_0.5i_ is the half-maximal voltage for inactivation, and *k_i_* the slope factor. Data are presented as mean ± SEM. Asterisks denote a significant difference from the WT control (*, *t*-test: p < 0.05). n: number of observations. *n.a.*: not applicable.

|  | | Activation | | | Inactivation (with Kvβ1.1) | | |
| --- | --- | --- | --- | --- | --- | --- | --- |
|  |  | V_0.5a_ (mV) | *k_a_* | n | V_0.5i_ (mV) | *k_i_* | n |
| **Monomer (co-expression)** | WT | -43.2 ± 2.0 | 4.4 ± 1.3 | 30 | -52.5 ± 3.3 | 4.4 ± 2.2 | 14 |
|  | WT+I262T (1:0.5) | -44.0 ± 3.9 | 3.8 ± 2.2 | 12 | *n.a.* | | |
|  | WT+I262T (1:1) | -37.1 ± 0.9* | 6.0 ± 0.4* | 17 | *n.a.* | | |
|  | WT+I262T (1:2) | -31.9 ± 2.7* | 6.9 ± 1.1* | 11 | *n.a.* | | |
|  | WT+I262T (1:5) | -19.2 ± 2.6* | 8.9 ± 1.5* | 8 | *n.a.* | | |
|  | WT+I262T (1:10) | -14.7 ± 4.1* | 9.2 ± 3.5* | 11 | *n.a.* | | |
|  | I262T | -9.6 ± 3.8* | 9.4 ± 0.7* | 19 | -33.0 ± 1.5* | 4.7 ± 1.8 | 24 |
| **Dimer** | WT-WT | -45.0 ± 1.3 | 3.9 ± 0.3 | 24 | -52.2 ± 2.0 | 5.0 ± 2.5 | 15 |
|  | WT-I262T | -32.2 ± 2.6* | 5.0 ± 1.7 | 27 | -42.7 ± 2.8* | 4.7 ± 3.0 | 10 |

***Supplementary Table S2. Kinetic parameters for “recovery from inactivation” and “cumulative inactivation” of human Kv1.1 WT or I262T channels.*** (related to Figure 4)

The time constant of inactivation recovery (τ_recovery_) were derived from one-exponential fit of the data points in the “current amplitude-interpulse duration” plot (see Figure 4C, right panel). The time constants of cumulative inactivation (τ_decay_) were determined from one exponential fit of the data points in the “current amplitude-test pulse number” plot (see Figure 4D, right panel). Data are presented as mean ± SEM. Asterisks denote a significant difference from the WT control (*, *t*-test: p < 0.05). Numbers of observations are shown in parentheses.

|  | | τ_recovery_ (sec) | τ_decay_ (pulse number) |
| --- | --- | --- | --- |
| (+) Kvβ1.1 | WT | 1.1 ± 0.1 (12) | 0.9 ± 0.1 (13) |
|  | WT-I262T dimer | 1.3 ± 0.1 (6) | 1.6 ± 0.1* (11) |
|  | I262T | 1.0 ± 0.1 (17) | 3.4 ± 0.1* (10) |

***Supplementary Table S3. Voltage-dependent activation and inactivation properties of human Kv1.4 channels in the presence of Kv1.1 WT or the I262T mutant.*** (related to Figure 5)

Steady-state activation and inactivation curves were generated by fitting normalized peak current amplitudes in response to different test pulse and prepulse potentials, respectively, with the Boltzmann function as described in Suppl. Table S1. Data are presented as mean ± SEM. Asterisks denote a significant difference from the WT control (*, *t*-test: p < 0.05). n: number of observations. *n.a.*: not applicable.

|  | | Activation | | | Inactivation | | |
| --- | --- | --- | --- | --- | --- | --- | --- |
|  |  | V_0.5a_ (mV) | *k_a_* | n | V_0.5i_ (mV) | *k_i_* | n |
| **Monomer** | Kv1.4 | -22.3 ± 2.3 | 20.2 ± 2.3 | 21 | -48.1 ± 5.2 | 3.9 ± 1.2 | 20 |
|  | Kv1.4+Kv1.1WT (1:1) | -22.8 ± 2.3 | 19.1 ± 2.3 | 22 | -48.7 ± 3.3 | 3.7 ± 0.3 | 19 |
|  | Kv1.4+Kv1.1I262T (1:1) | -16.0 ± 1.6* | 16.7 ± 1.6 | 26 | -32.4 ± 2.4* | 7.6 ± 3.7 | 27 |
| **Dimer** | Kv1.4-Kv1.1 WT | -23.1 ± 2.0 | 17.4 ± 1.9 | 20 | -44.4 ± 4.7 | 4.9 ± 1.9 | 23 |
|  | Kv1.4-Kv1.1I262T | -11.9 ± 1.8* | 16.4 ± 1.8 | 16 | -31.4 ± 1.8* | 6.0 ± 3.4 | 17 |
|  | Kv1.4-Kv1.1WT  (+ Kvβ1.1) | *n.a.* | | | -50.7 ± 3.9 | 4.8 ± 1.5 | 11 |
|  | Kv1.4-Kv1.1I262T  (+ Kvβ1.1) | *n.a.* | | | -37.9 ± 3.6* | 6.2 ± 2.8 | 9 |

***Supplementary Table S4. Kinetic parameters for the “recovery from inactivation” and the “cumulative inactivation” of human Kv1.4 channels in the presence of Kv1.1 WT or the I262T mutant.*** (related to Figure 5)

The time constants of inactivation recovery (τ_recovery_) and cumulative inactivation (τ_decay_) were determined as described in Supplementary Table S2. Data are presented as mean ± SEM. Asterisks denote a significant difference from the WT control (*, *t*-test: p < 0.05). Numbers of observations are shown in parentheses.

|  | | τ_recovery_ (sec) | τ_decay_ (pulse number) |
| --- | --- | --- | --- |
| (-) Kvβ1.1 | Kv1.4-Kv1.1 WT dimer | 0.54 ± 0.03 (13) | 11.8 ± 0.1 (14) |
|  | Kv1.4-Kv1.1 I262T dimer | 0.66 ± 0.02 (17) | 19.8 ± 0.4* (19) |
| (+) Kvβ1.1 | Kv1.4-Kv1.1 WT dimer | 1.9 ± 0.2 (10) | 2.16 ± 0.03 (14) |
|  | Kv1.4-Kv1.1 I262T dimer | 1.4 ± 0.1 (12) | 3.94 ± 0.04* (14) |

***Supplementary Table S5. Window current parameters for human Kv1.1 and 1.4 channels.*** (related to Figure 6)

As illustrated in Figure 6, the window current is defined by the triangular area underneath the overlap of activation and inactivation curves. The peak voltage refers to the membrane potential at which the apex of window current triangle is located. The area of window current triangle was determined by integration. For each construct, the area of window current was normalized with respect to that of the corresponding WT control.

| (+ Kvβ1.1) | Peak voltage (mV) | Normalized area |
| --- | --- | --- |
| Kv1.1 WT | -47.6 | 1.0 |
| Kv1.1 I262T | -24.7 | 0.7 |

| (+ Kvβ1.1) | Peak voltage (mV) | Normalized area |
| --- | --- | --- |
| Kv1.1 WT-WT dimer | -47.9 | 1.0 |
| Kv1.1 WT-I262T dimer | -37.2 | 0.8 |

|  | Peak voltage (mV) | Normalized area |
| --- | --- | --- |
| Kv1.4-Kv1.1 WT dimer | -38.5 | 1.0 |
| Kv1.4-Kv1.1 I262T dimer | -25 | 1.4 |

***Supplementary Figure S1. Representative current traces from Xenopus oocytes co-expressing Kv1.1 and Kvβ subunits.*** (related to Figure 2A)

Kv1.1 WT, I262T, or equal-molar co-expression [WT+I262T (1:1)] was co-expressed with Kvβ1.1 ***(A)*** or Kvβ2 ***(B)*** (molar ratio 1:5) in *Xenopus* oocytes. The voltage protocol is the same as that in Figure 1A. The external solution contains 3 mM KCl.


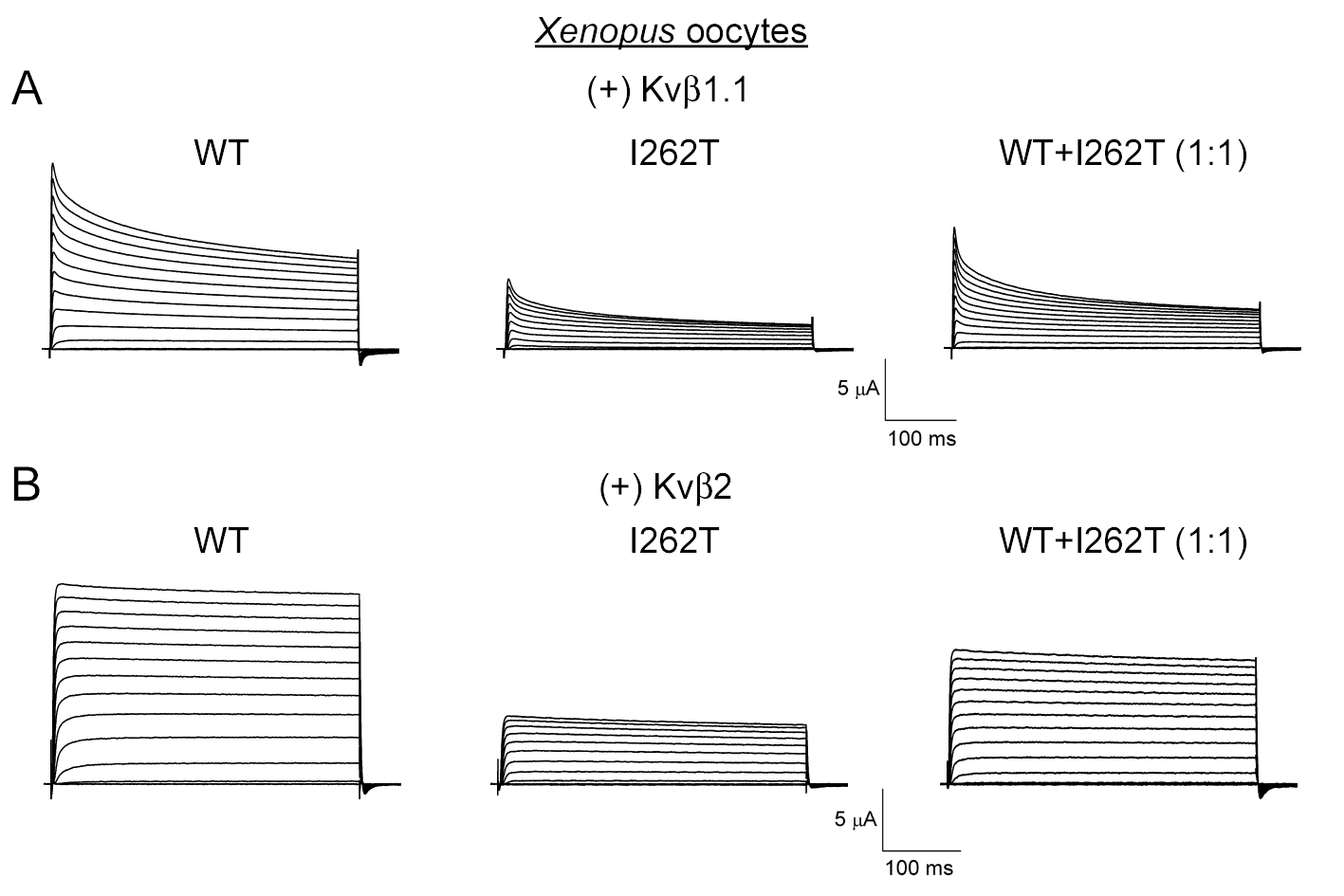


***Supplementary Figure S2. Representative Kv1.1 current traces in HEK293T cells.*** (related to Figure 2BCD)

Kv1.1 WT, I262T, or equal-molar co-expression [WT+I262T (1:1)] was expressed in the absence ***(A)*** or presence of Kvβ1.1 ***(B)*** or Kvβ2 ***(C)*** (molar ratio 1:5) in HEK293T cells. ***(D)*** Functional expression Kv1.1 WT-WT dimer or WT-I262T dimer in HEK293T cells. The voltage protocol is the same as that in Figure 1A. The external solution contains 5 mM KCl.


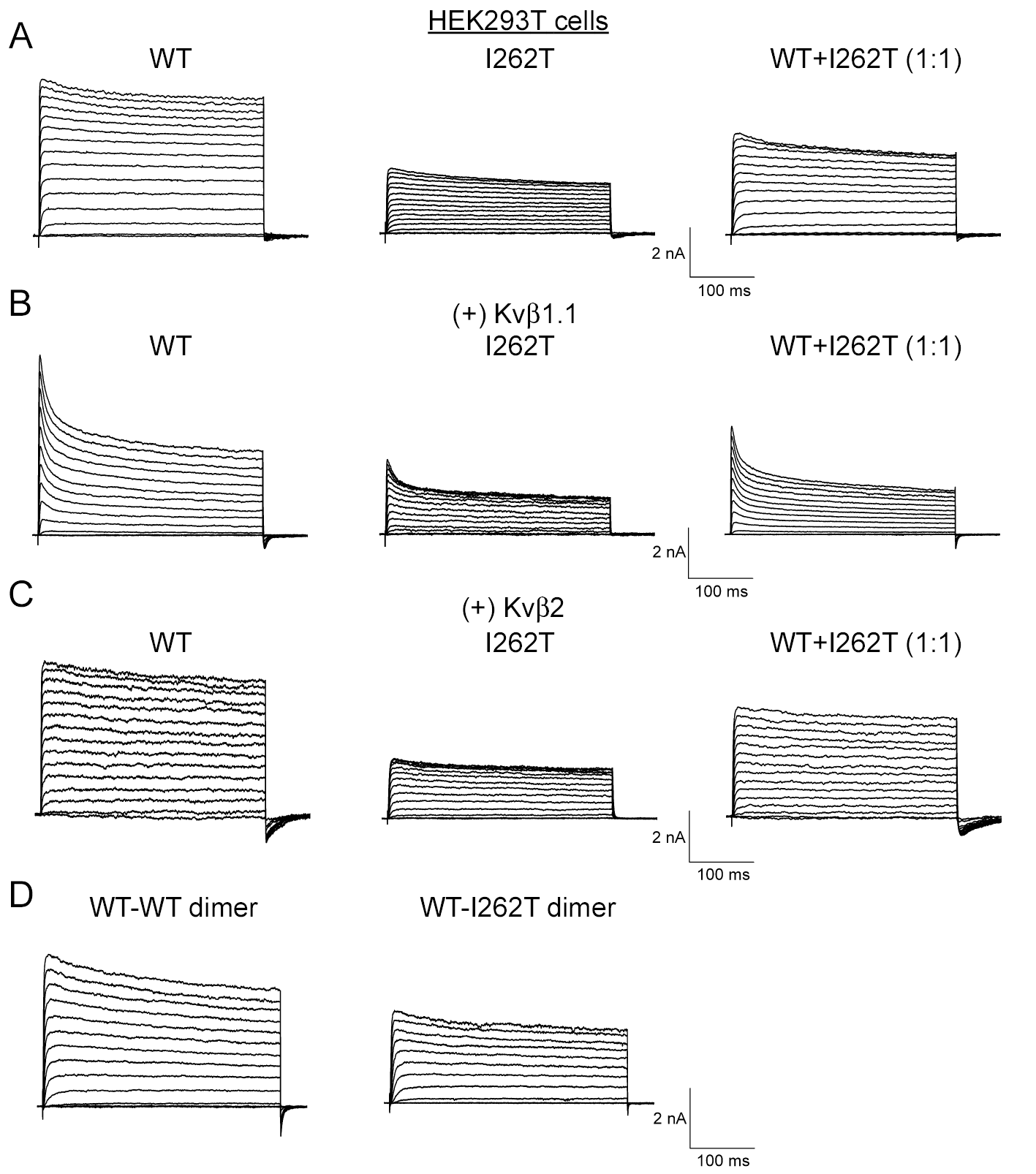


***Supplementary Figure S3. Cycloheximide chase for 6 hours.*** (related to Figure 3B)

***(A)*** Representative immunoblots showing the protein turn-over time course of Myc-Kv1.1-WT and Myc-Kv1.1-I262T in the presence of different treatment durations of 100 μg/ml cycloheximide (CHX). The gels were run under the same experimental conditions. ***(B)*** *(Left)* Quantification of Kv1.1 protein degradation time course. Protein densities were standardized with respect to the cognate GAPDH signal, followed by normalization to the corresponding control at 0 hr. Data points represent the average of 4-5 independent experiments. *(Right)* Linear-regression analyses (solid lines) of the semi-logarithmic plot of Kv1.1 degradation time course. The protein half-life derived from the linear regression lines are about 4.1 and 2.8 hrs for the WT and I262T, respectively. ***(C)*** Statistical analyses of the protein half-life between the WT and I262T. The protein degradation time course determined from each transfection was individually plotted on a semi-logarithmic scale for linear-regression analyses. The numbers in parentheses refer to the total number of transfections performed in the 4-5 independent experiments. The mean protein half-life for the WT and I262T are about 4.7±0.6 and 2.7±0.4 hrs, respectively. The asterisk denotes a significant difference from the WT control (*, *t*-test: p < 0.05).


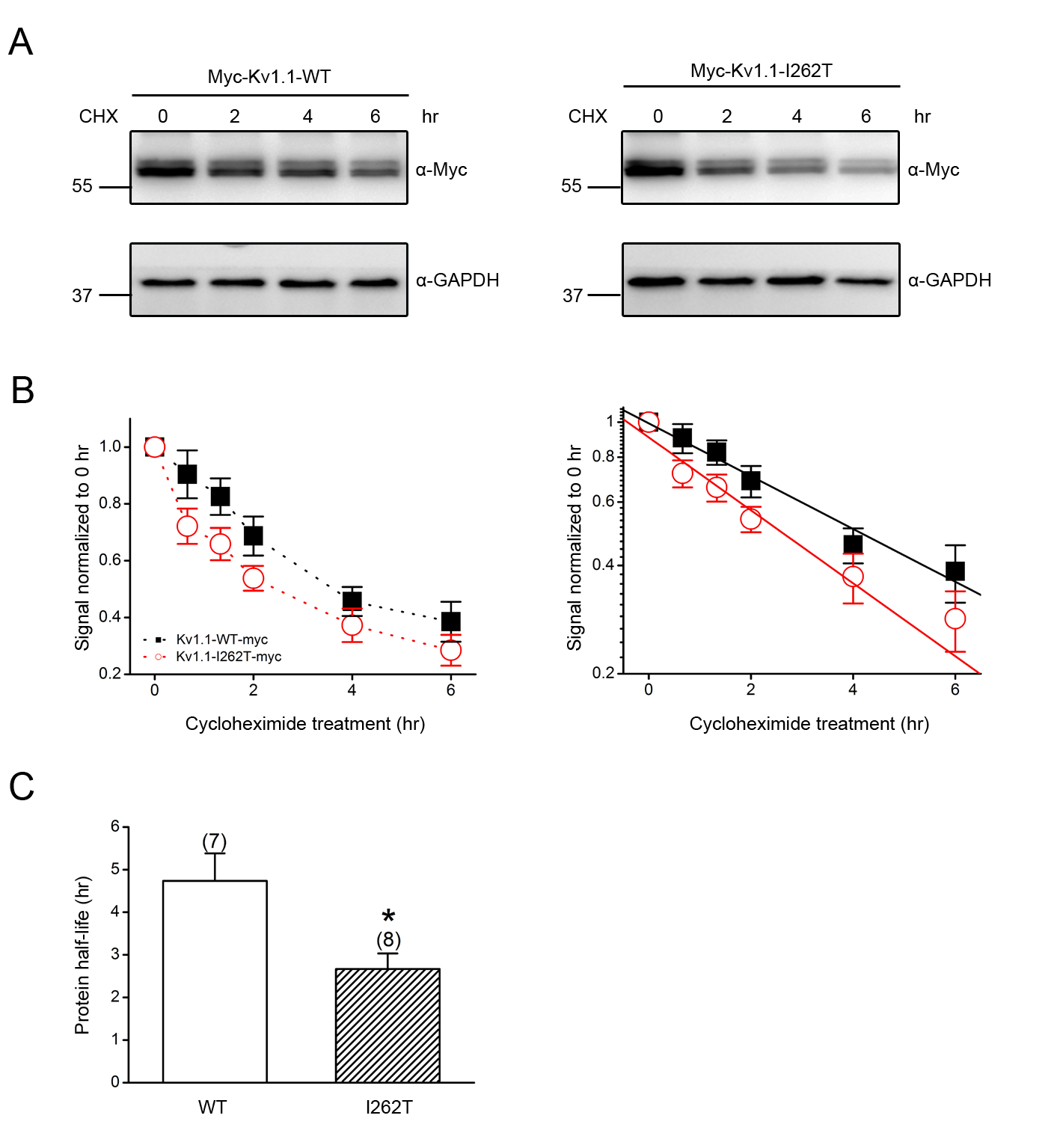


***Supplementary Figure S4. Co-expression of Kv1.2 and Kv1.1 I262T in Xenopus oocytes.***

***(A)*** Kv1.2 does not appear to affect the defective expression of I262T. *(Left)* Representative current traces of equal-molar co-expression of “Kv1.2 and Kv1.1 WT” (Kv1.2+Kv1.1 WT) or co-expression of “Kv1.2 and Kv1.1 I262T” (Kv1.2+Kv1.1 I262T) in the 3-mM KCl bath solution. The voltage protocol is the same as that in Figure 1A. *(Right)* Normalized current amplitudes (at +60 mV) for the two co-expression conditions. ***(B)*** I262T changes the voltage-dependence of Kv1.2/Kv1.1 co-expression. *(Left)* Functional co-expression of “Kv1.2+Kv1.1 WT” or “Kv1.2+Kv1.1 I262T” in the 60-mM KCl bath solution. *(Right)* Steady-state activation (*P_o_*–V) curves derived from isochronal tail currents at -90 mV in response to various test pulse potentials. Based on curve fitting of the data points with the Boltzmann function, the half-maximal voltage (mV) and the slope factor for “Kv1.2+Kv1.1 WT” are -34.5 ± 1.3 and 4.2 ± 0.2 (n = 8), respectively. The half-maximal voltage and the slope factor for “Kv1.2+Kv1.1 I262T” are -25.0 ± 1.5 and 5.7 ± 0.5 (n = 8), respectively.


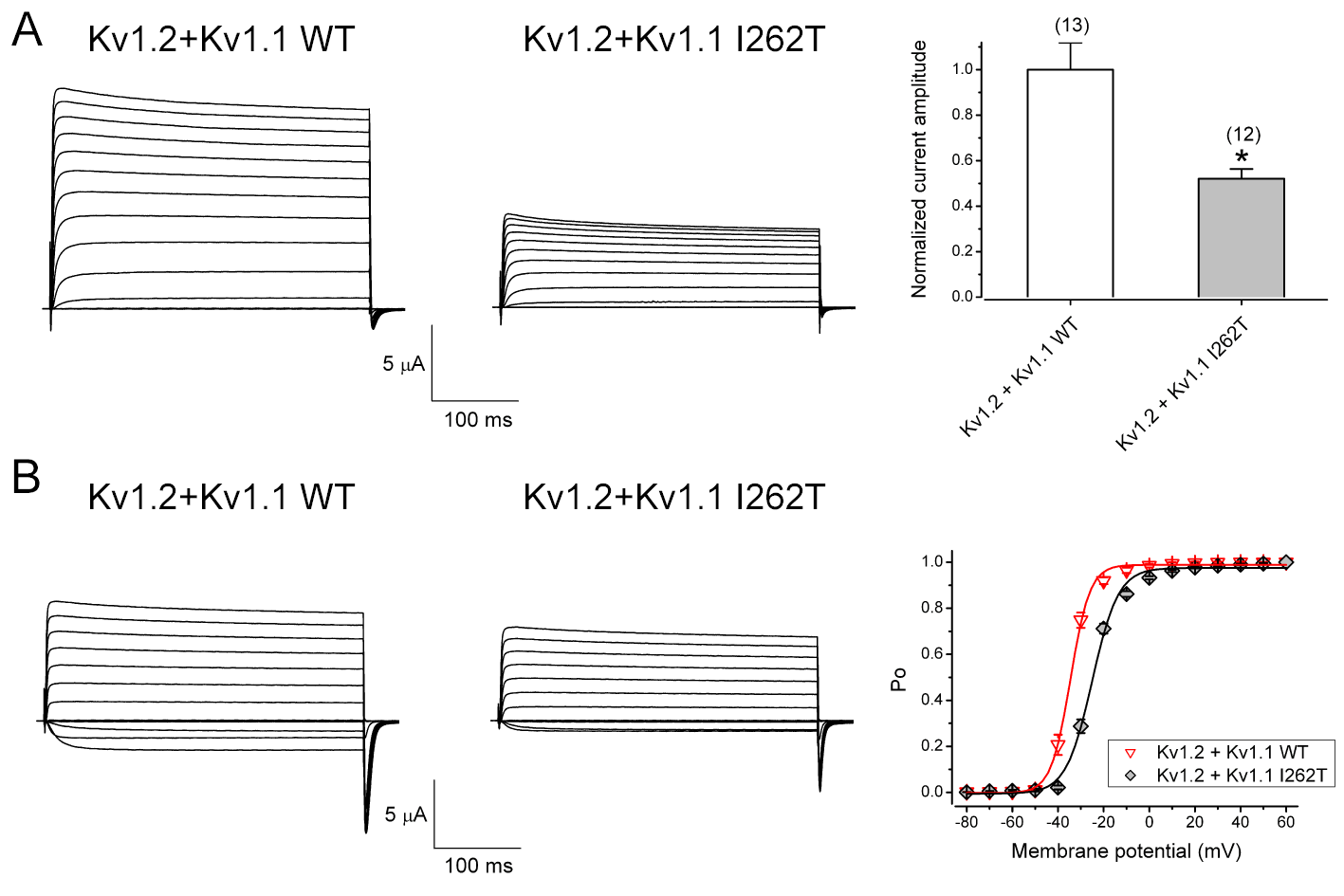


***Supplementary Figure S5. Uncropped images of the immunoblots presented in the main figures.*** (related to Figure 3)


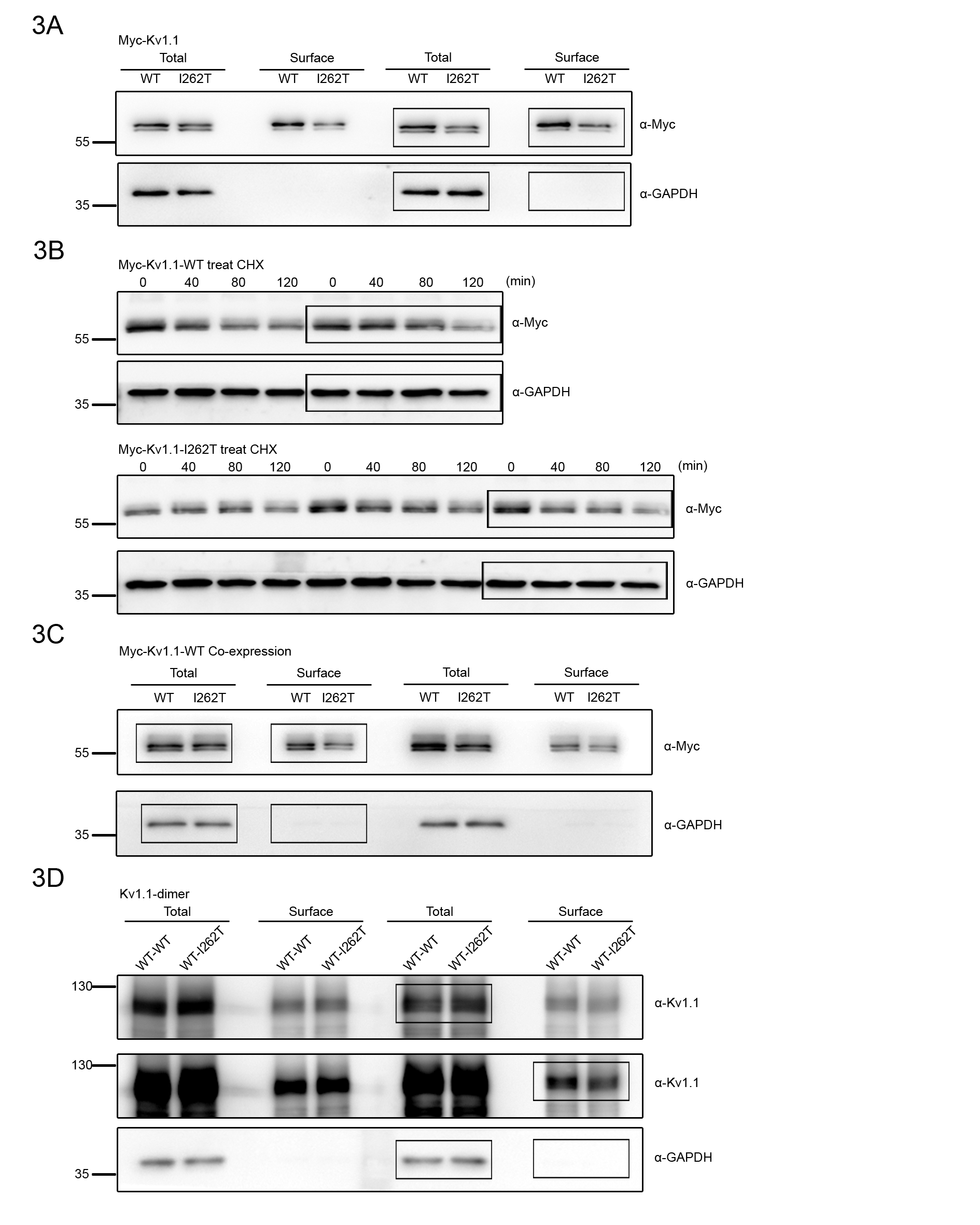

Supplement: Supplementary Information [file srep19378-s1.doc]
